# The complete mitochondrial genome of grey plover *Pluvialis squatarola* (Charadriiformes, charadriidae)

**DOI:** 10.1080/23802359.2020.1787892

**Published:** 2020-07-11

**Authors:** Jingjing Ding, Ruen Qian, Deyun Tai, Wenjia Yao, Chaochao Hu, Qing Chang

**Affiliations:** a Jiangsu Academy of Forestry, Jiangsu, Nanjing, China; b Jiangsu Key Laboratory for Biodiversity and Biotechnology, College of Life Sciences, Nanjing Normal University, Jiangsu, Nanjing, China; c Analytical and Testing Center, Nanjing Normal University, Jiangsu, Nanjing, China

**Keywords:** *Pluvialis squatarola*, mitochondrial genome, Charadriiformes

## Abstract

The complete mitochondrial genome of grey plover *Pluvialis squatarola* was obtained by next-generation sequencing. The circular genome was 16,860 bp in length, consisting of 13 protein-coding genes, 22 transfer RNA genes, 2 ribosomal RNA genes, and a control region. The overall nucleotide composition was A: 30.9%, T: 23.4%, C: 31.6%, G: 14.1%. Nine genes were encoded on the light strand, and the remaining 28 genes were encoded on the heavy strand. Most of the PCGs began with the ATG as the start codon, and four kinds of termination codons were used in this mitogenome. This study improves our understanding of the mitogenomic characteristics and its phylogenetic relationships within Charadriiformes.

The grey plover *Pluvialis squatarola* (Charadriiformes, Charadriidae) is full migratory with an extremely large distribution range, which has short and thick beak, but wings and belly are white. Recent studies of *P. squatarola* place emphasis on habitat ecology and migration routes or strategies (Exo et al. 2019). Nonetheless, the basic genetics data of *P. squatarola* is unclear. In this study, we sequenced the complete mitogenome of *P. squatarola* to better understand the mitogenomic characteristics and its phylogenetic relationships in Charadriiformes.

The muscle specimen of *P. squatarola* was collected from Xiaoyangkou, Nantong, Jiangsu Province, China (32°30′12.48″N, 121°11'04.59″E). The voucher specimen was preserved in absolute ethanol, which was stored at −20 °C in our laboratory at Nanjing Normal University, Jiangsu, China (specimen voucher: NJNU-Psqu04). Total DNA was extracted with standard phenol-chloroform methods (Sambrook et al. 1989). The sequencing libraries with average insert sizes of approximately 300 bp were prepared, and then sequenced as 150 bp paired-end runs (about 8 Gb raw data) on the Illumina HiSeq 2000 platform (Illumina, San Diego, CA, USA). The software of Geneious 9.1.4 was used for sequence quality analysis, data trimming, and *de novo* assembling (Kearse et al. 2012). The locations of the protein-coding genes, ribosomal RNAs (rRNAs), and transfer RNAs (tRNAs) were predicted using MITOS Web Server and identified by alignment with other mitogenome of Charadriiformes species (Bernt et al. 2013).

The circular mitogenome is 16,860 bp in length with 13 protein-coding genes (PCGs), 2 ribosomal RNAs (12S rRNA and 16S rRNA), 22 transfer RNA genes, and a non-coding region. The annotated mitogenome of *P. squatarola* has been deposited in GenBank (MT561267). The overall nucleotide composition was A: 30.9%, T: 23.4%, C: 31.6%, G: 14.1%. Among the 37 genes, nine genes (tRNAGln, tRNAAla, tRNAAsn, tRNACys, tRNATyr, tRNASer, ND6, tRNAPro and tRNAGlu) were encoded on the light strand, and the remaining 28 genes were encoded on the heavy strand. The total length of 13 protein-coding genes is 11,394 bp, accounting for 67.6% of the complete genome. Most of the PCGs began with the ATG as the start codon.

We performed a phylogenetic analysis based on mitochondrion complete genome of the available mitogenome sequences of Charadriiformes and two other species (*Phodilus badius*, *Gallus gallus*), using maximum likelihood (ML) algorithm in MEGA X (Kumar et al. 2018). The mitogenomes sequences was aligned using Muscle in MEGA X, and subsequently all positions containing gaps and missing data were edited and eliminated (Kumar et al. 2018). Finally, there were a total of 15,282 used for phylogenetic analyses.with the general time-reversible (GTR) model and the lowest BIC scores (Bayesian Information Criterion). Evolutionary analyses were conducted in MEGA X. The phylogenetic analysis (Figure 1) resolved three well-supported clades of Charadriiformes, indicating great divergence within the Charadriiformes. This study improves our understanding of the evolution of mitochondrial DNA in Charadriiformes.

**Figure 1.**
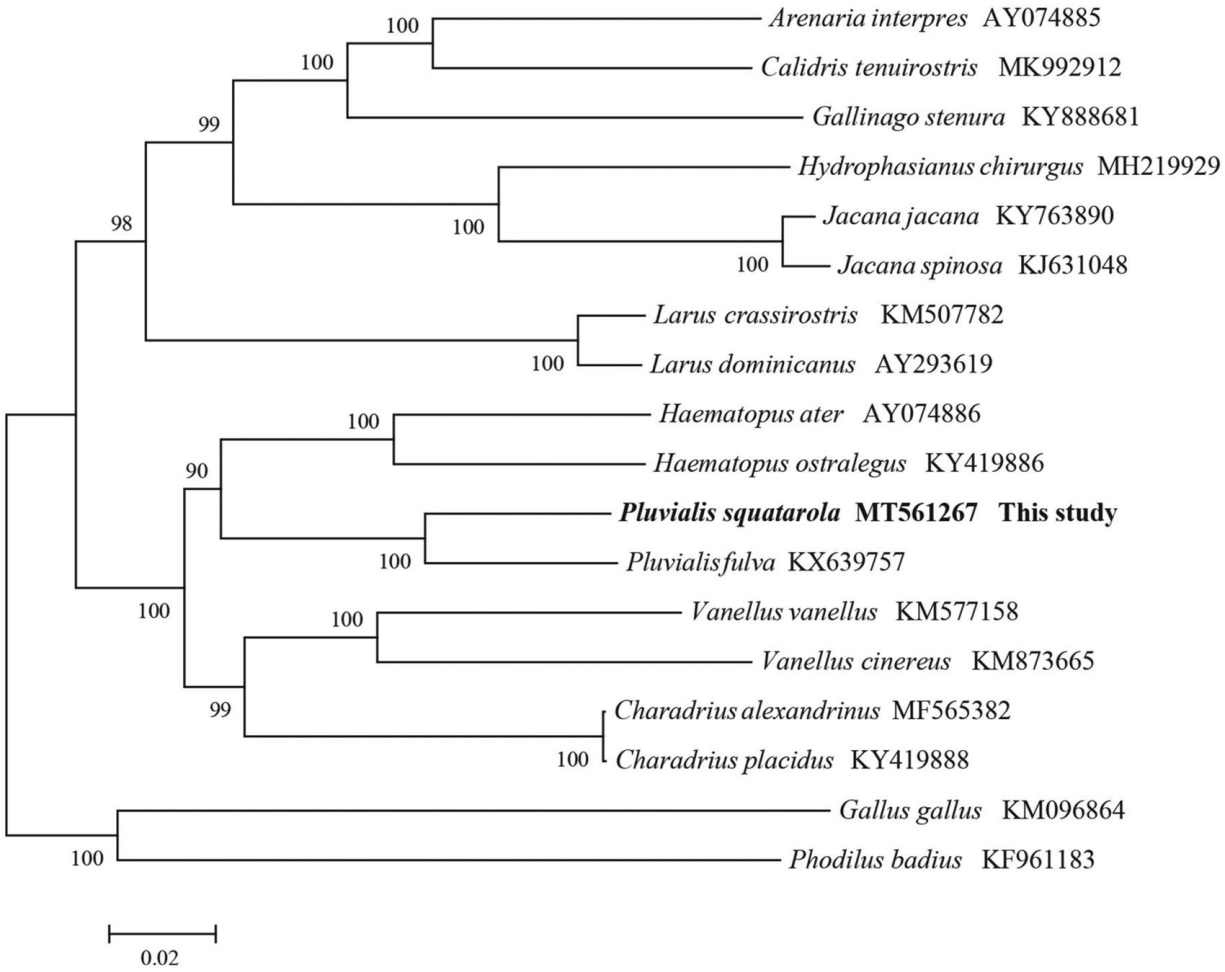
Phylogeny of *P. squatarola* and closely related 15 mitochondrial sequences constructed using the maximum likelihood (ML) method by analyzing mitochondrion complete genome. Numbers above each branch is the ML bootstrap support.

## Data Availability

The data that support the findings of this study are openly available in National Center for Biotechnology Information (NCBI) at https://www.ncbi.nlm.nih.gov/, reference number MT561267.
